# Family caregivers’ accounts of caring for a family member with motor neurone disease in Norway: a qualitative study

**DOI:** 10.1186/s12904-016-0097-4

**Published:** 2016-02-24

**Authors:** Sverre Vigeland Lerum, Kari Nyheim Solbrække, Jan C. Frich

**Affiliations:** Institute of Health and Society, University of Oslo, P. O. Box 1089, N-0318 Oslo, Norway

**Keywords:** Motor neurone disease, Amyotrophic lateral sclerosis, Caregivers, Palliative care, Qualitative research, Care work, Meaning, Health service refusal, Proactive

## Abstract

**Background:**

Motor neurone disease (MND) is a progressive neurological disease causing muscle wasting, gradual paralysis, respiratory failure. MND care is demanding, complex and involves a variety of care tasks. Family members may experience significant and enduring strain. We conducted a qualitative study to understand more about family caregivers’ work and sense of responsibility, exploring family caregivers’ accounts of caring for a family member with MND.

**Methods:**

We recruited and interviewed a total of 25 participants from Norway, including 17 current and eight bereaved family caregivers. Drawing on theories of care by Corbin and Strauss, we analysed the data by a theoretical reading of the material to identify different types of care work.

**Results:**

We found that caregivers were engaged in five lines of care work that could be parallel or closely interconnected: i) immediate care work; ii) seeking information and clarity about the disease; iii) managing competing obligations; iv) maintaining normality; and v) managing external resources and assistance. Caregivers’ priorities were shaped by their interactions with the person with MND, available assistive devices, the development of the illness, and utilisation of paid care. Care work had a symbolic and moral meaning for caregivers, and was associated with self-worth and respect from others. Caregivers tried to balance their own expectations and others’ expectations without being overwhelmed by care work.

**Conclusions:**

A changing and potentially chaotic situation for family caregivers may compromise their capacity to utilise supportive services. Using the lines of work as a framework to assess caregivers’ preferences and priorities, health professionals may tailor assistance and support to family members caring for persons with MND.

## Background

Family caregivers play an important role in palliative care through managing long-term illness in the family [1–3]. Motor neurone disease (MND), also known as amyotrophic lateral sclerosis (ALS), causes muscle wasting, breathing and swallowing difficulties, respiratory failure and may involve cognitive impairment [4, 5]. The mean age for onset of MND is in the late fifties [4]. There is no known cause or cure for the disease, but several interventions may improve quality of life and prolong life expectancy beyond the average of 2–4 years [4].

There is considerable variation in the clinical course of MND [5], and as the disease unfolds care tasks rapidly change, and decision-making has been described as “ongoing change and adaptation” [6]. Health professionals, people with MND and caregivers may alternate between seeing MND as a terminal and a chronic illness [7]. Preferences for assistance may change during the course of the illness [8], and may fluctuate between alleviating family concerns and at the same time needing family assistance, often depending on when MND occurs in the life-course [9]. The amount of care work may grow beyond the capacity of a single individual.

Family members caring for those with MND may experience significant and enduring strain due to rapid unfolding of the illness [10–12] and caregiver needs are not necessarily the same as the needs of people with MND [13]. Family caregivers’ work can be up to 15 h a day in the late phase of the illness [14], and they may downplay their own need for sleep, recreation or exercise [15]. Caregivers may feel they have responsibility for the survival of the person with MND [10] and some persons with MND may perceive that their life is already over [16]. The literature is not consistent as to whether the person with MND’s functional decline increases a caregiver’s strain and burden, but the cognitive and behavioural status of the person with MND seems to do so [17]. Several barriers to utilisation of professional assistance have been noted. Paid carers may disrupt a sense of normality and privacy at home, and family caregivers may feel a strong sense of duty towards the person with MND [18]. Family caregivers may also refrain from services because they perceive the disease progression as rapid, and think they can manage on their own for the time that is left [7]. Some may postpone initiating service use, having high trust in primary care believing that advanced services are readily available, which, however, creates a feeling of betrayal if faced with a long waiting time [19]. Studies argue that refusing services may be a way of exerting control over the disease and the losses it represents [20, 21]. Users may postpone utilising supportive services until exhaustion [15], and until the situation is so severe that it no longer feels like a choice [18]. Some suggest that health professionals should attempt to identify struggling caregivers early [15], while others propose that persons with MND should engage with services “on their own terms” [20]. Hence, when professionals initiate services, one may not assume the domestic space to be free of conflict, complicating the issue of whether those at home or the health services should be the ones initiating service utilisation. While family caregivers experience much strain, they may have several reasons to refuse or to be reluctant towards utilising services.

Through developing theory about MND care work, we argue that a more nuanced understanding of caregiver work may help family caregivers to articulate care needs, and health professionals to fine-tune initiation of services. We draw on Corbin and Strauss’s [22] classic theory of care work, which is based on studies of an array of different diagnoses. When studying several illnesses, two different conditions could be regarded as typical chronic illnesses, but each condition could entail different types of tasks. To solve this analytical problem, Corbin and Strauss conceptualise handling illness along lines of work. Lines of work comprise a level of abstraction above tasks, allowing comparison even though the tasks may be different or changing. The three lines of work are: 1) illness work, work directly related to or directed at the illness, 2) biographical work or identity work, work to accommodate a new life with illness into one’s sense of self and biography, and 3) everyday work. Everyday work is work not directly related to illness management, such as occupational work, raising children or shopping for groceries. As tasks are rapidly changing in the MND context, we find the notion of work to be a useful concept to understand MND caregiving. However, providing practical advice in the MND context, the original theory easily becomes too abstract. Hence, we want to increase the applicability of this theory by conceptualizing findings specifically towards MND, rather than chronic illness in general.

In order to untangle the complexities in MND care we have used Corbin and Strauss’s theory [22] as a starting point to develop new theory aimed at the specific MND context. Further, characteristic for the Norwegian setting is a generous welfare state, where caregivers and persons with MND have strong legal rights to paid care and assistive devices, with little direct financial cost [23]. We had an interest in understanding caregivers’ expectations regarding performance of care work and utilisation of services. With this theoretical and contextual backdrop we focus on two research questions: 1) What lines of work are caregivers engaged in? 2) What social norms are these lines of work embedded in? By understanding more about caregivers’ work and sense of responsibility, we hope to contribute to enhanced collaboration and an increased uptake of supportive services.

## Methods

### Participants

In 2011–2012 the first author interviewed a total of 25 participants: 17 active family caregivers, and 8 bereaved family caregivers (Table 1). In five cases family caregivers wanted the person with MND to be present during the interview, which was respected. In one interview three family members wanted to participate, and the 25 family caregiver participants comprise 23 interviews. Among the caregivers, some had years of experience of living with the condition, while others had received the diagnosis a few months before. Participants were recruited through health professionals, who were coordinators at specialised MND-clinics in hospitals, in three different vicinities. They explained the purpose of the study and asked caregivers for permission to pass on their contact information to the first author, who informed them about the study and obtained participants’ written informed consent.

**Table 1:** Characteristics of participants who were interviewed

Characteristic	*n* = 25
Group	
Bereaved family caregiver	8
Current family caregiver	17
Age	
20–29	2
30–39	2
40–49	7
50–59	11
60–69	6
70–79	2
Gender	
Male	12
Female	18
Educational status	
> 3 years of higher education	12
< 3 years of higher education	6
No higher education	12

### Interviews

The first author conducted the interviews. Most interviews took place in the caregivers’ homes, with some in public places, as chosen by the participants. The interviews lasted between 1 and 2 h, and were digitally recorded and transcribed verbatim. The interviews had two parts. The first section had a narrative approach, where the participants were encouraged to talk about their experiences with MND in their own words, for as long as they wanted, starting from the first time they suspected something to be unusual. The talk was structured along the timeline, and the interviewer encouraged participants to stay on topic and elaborate on their understanding of MND and experiences with caring for an individual with MND. The latter part of the interviews was semi-structured, aimed at being used as a checklist if something was omitted in the first part. The pre-defined topics included “establishing the diagnosis”, “the role of the caregiver”, “experiences with primary health care” and “experiences with the hospital”. The interviews for this study were part of a larger project focusing on coordination of care for people with MND [7]. The interview guide was developed during the first 8 interviews with bereaved caregivers, to get an overview of the illness trajectory from beginning to end. The semi-structured of the guide part was developed for the interviews with caregivers to correspond with interviews with primary care staff and hospital staff working with MND. The full interview guide was seldom used actively in the interviews, rather it was a preparatory tool before each interview, and it functioned as a checklist to ensure that relevant themes had been reflected upon and to round up the interview.

### Analysis

All three authors routinely met during the data collection period. Transcripts were read by all authors in order to obtain an overall impression of the material, and identify themes to explore in subsequent interviews with new participants. The analysis consisted of several steps. Initially, the first author wrote summaries of interviews, and each summary followed the same structure focusing on the family caregivers’ relationship to: 1) the person with MND, 2) primary health care, 3) the hospital and 4) other activities not directly related to MND. The summaries were of varying length, but averaged four pages.

The next step followed Kvale and Brinkmann’s [24] analytic approach for interpretation of meaning, interpreting beyond what is directly said in the interviews. We did a theoretical reading [24] using Corbin and Strauss’s [22] notion of ‘work’ to ask analytic questions and highlight common themes in the memos. In this analytic work the material was discussed against original three lines of work, and we found that important aspects of the participants’ efforts received limited attention; such as regulating the time and energy of care work spent in physical proximity to the person with MND, as well as the various tactics to handle excesses of information on the internet. A typology of lines of care work was developed based on a small selection of the summaries (4) and the typology was then applied and refined on the rest of the material. All authors discussed typology in the following step, resulting in adjusting the typology and further validation and refinement against the original summaries and transcripts, was needed. Hence, the analytic process involved several rounds of presenting typology suggestions, which then was applied and refined against memos and the original transcripts. In the subsequent writing-up phase, we chose to present a descriptive section focusing on the typology, and another section focusing on expectations. The analysis was performed on transcripts in Norwegian. When the analysis phase was finished, illustrative quotes were translated from Norwegian to English. All authors checked the quality of the final translation.

### Ethics

The study was submitted to the Regional Committees for Medical and Health Research Ethics in Norway (ref: 2010/3334), which found that the project was exempt from review. The project was approved by the Norwegian Social Science Data Services (ref: 26498/3/KS).

## Results

### Five lines of care work

We found that caregivers were engaged in five lines of care work that could be parallel or closely interconnected, and that their priorities about different kinds of work changed continuously during the course of the illness (Table 2).

**Table 2:** Lines of care work

Line of work	Characteristics
Immediate care work	Work in physical proximity to the person in need of care, to replace functionality or provide a sense of safety.
Seeking information and clarity	Work to handle information about the illness, prognostic outlook and ways to manage the situation.
Managing competing obligations	Work to accommodate other obligations than those related to the illness
Maintaining normality	Work to provide a sense of normality, such as remain in paid employment or sustain a social life.
Managing external resources and assistance	Work to handle and incorporate community health workers, friends and family and assistive devices at home.

#### Immediate care work

Immediate care work required physical proximity to the person with MND, and involved assistance to replace lost functions, such as repositioning arms and legs or turning a page in a book. Another aspect was being available and physically present for safety reasons, monitoring life-support equipment, e.g. percutaneous endoscopic gastrostomy (PEG) or respiratory support. Family caregivers were often the only backup if professionals did not show up. Immediate care work put a strain on caregivers, as they often had to be available day and night. This work involved physical proximity, but was often psychologically motivated. A caregiver described her role as being a psychological guarantor of safety:

*“I have, in a sense, been a mini-psychologist. She* [the person with MND] *is very safe when I’m around. That’s what she says, ‘you are my safety’.”* (Participant 9, family caregiver, 9 years since first observed symptom)

The personal relationship between the caregiver and the one who was cared for gave meaning to the immediate care work. According to some caregivers and persons with MND, the personal and relational nature of this work could create a barrier to utilising paid personnel, as a stranger performing the work would weaken its personal and relational dimension. Immediate care work was important throughout the trajectory, but the need to adapt to loss of functions increased as the disease unfolded, causing the amount of this type of work to accumulate.

#### Seeking information and clarity

Caregivers were engaged in seeking information and clarity about MND. Yet, while some participants found that information gave a sense of control and prepared them for the future, others underlined the importance of embracing the moment and avoiding too much information. Caregivers could screen the information they found before sharing it with the person with MND. Some caregivers looked for information to challenge the diagnosis, by exploring other possible and treatable diseases causing the symptoms, or searching for a cure. A family caregiver recounted his experiences:

“*Sure, I have read* [the information leaflets]*, among other things. […] And if she* [the person with MND] *is wondering about something, or is feeling bad 1 day, I can say: ‘No, it has nothing to do with* [MND]*, you’re just in bad shape, or you have a cold or something like that’ […] It is much better that she’s living the life she wants to live, and then I can carry some of the burdens for her.”* (Participant 24, family caregiver, 2 years since first observed symptom)

The various information strategies had implications for caregivers’ handling of different types of loss, such as coping with functional decline, coming to terms with the prognostic outlook, and death. This line of work typically implied much effort in the early phases of the illness trajectory, before and after being diagnosed. Typically, seeking information and clarity would diminish when coming to terms with the condition, and other lines of work would become more prominent. In some cases, gaining in-depth knowledge of the condition and seeking a cure by traditional or alternative means became an enduring effort, lasting the entirety of the trajectory and beyond.

#### Managing competing obligations

This line of work involved accommodating other obligations than those related to MND into everyday life; such as earning money, shopping for groceries or taking care of other family members. While caring for his brother with MND, one participant experienced the death of his father:

*“*[My father] *had a lot of those silent heart attacks, and everything just turned into chaos with him, when all this with* [my brother with MND] *happened. So you could say, at that time, I had two of them* [persons in need of care]*.”* (Participant 4, bereaved caregiver, trajectory lasted 4 years from first observed symptom)

Some family caregivers were responsible for children as well as parents, in addition to having needs of their own. Managing competing obligations, such as changing relationships within a family, involved time and effort. As the illness developed, a core issue was how to prioritise immediate care work, which would grow more and more demanding, against the other lines of work. Prioritisation of immediate care work often depended on whether the condition was considered terminal, and a sense of this being the final time together. A longer timeline made it easier to spend time and energy on other activities.

#### Maintaining normality

Effort was required to maintain a sense of normality, remaining in paid employment or sustaining a social life, which emphasised by most participants. As one bereaved caregiver put it:

*“And that was the challenge. To try to live as normally as possible, without creating more problems than those that already existed.”* (Participant 5, bereaved caregiver, trajectory lasted 3 years from first observed symptom)

We found that various strategies to maintain normality changed during the course of the illness. Families established arenas for common activities that gave a sense of normality, such as family meals, listening to music or watching movies. Utilising assistive devices played a key part in adapting to new situations, enhancing independence and creating a sense of normality. One way of establishing normality was to deliberately cut down on immediate care work, as when the person with MND suggested that the family caregiver spend time on usual activities as he/she did before the illness. On the other hand, immediate care work could also contribute to a sense of normality in itself, for example by refusing supportive services to enjoy privacy and intimacy. Working to maintain a sense of normality was important for caregivers, and this could start before a diagnosis was given, often while waiting for hospital consultations which might confirm a suspected serious illness.

#### Managing external resources and assistance

There were several types of external resources available to the caregivers, including community health workers, friends and family, Norwegian social insurance benefits or private insurance. Among the actors outside the home, primary care was a main player, as MND entailed a legal entitlement to care. Hence, routinely when MND was diagnosed, primary care officials assessed the situation at home, mapped care needs and initiated supportive measures based on the assessment. All participants had contact with paid caregiver support, but the amount of paid caregiver hours varied from zero to tens of hours per week. Primary care assessments continued throughout the trajectory, to adjust service delivery. External resources were available with little financial cost, but still demanded considerable time and energy on the caregivers’ part. Utilising external resources involved active work and effort, such as personal attendance at coordination meetings, learning to use assistive devices, participating in hospital consultations, making phone calls or arranging for home visits. Additionally, family caregivers could build alliances with preferred health professionals in order to achieve desired goals. This type of coordination work was ongoing as the care needs changed over the course of the trajectory. One participant described obtaining PEG nutrition in this way:

[The first time] *the hospital ordered the PEG food. It arrived at our door the day after. The next time, when we started to run out* [of food]*, I called* [the company providing the food]*. I ordered, and everything went well. The second time I called, they* [the company] *asked me if I could call social services, ‘because they hadn’t got any papers approving that she should have the PEG food.* […] *And when I called the social services…*[…] *Well, first they asked if they could talk to her* [the person with MND]*. “No, sorry, she only talks through a machine, so…,” I said. “Well, are you the proxy to formally talk on her behalf?” they asked. “I have been married to her for 20 years,” I said. “Okay, but we need to send you a form to sign,” to make me the proxy. But then I said, “I haven’t installed this PEG, they did it over at the hospital.” But it didn’t help, I had to fill in the form. There was nothing else to do but to hang up on them. But I got the food, finally, because I called them up later.* (Participant 14, family caregiver, 2 years since first observed symptom)

As the person with MND was unable to take food orally, it was critical to have a supply of PEG nutrition at home at all times. Dependence on external institutions, and this critical type of time constraint, was typical of the care needs the family caregivers handled. The quote above illustrates several forms of coordination activity, demanding time and energy from family caregivers. Further, even though supportive services were needed, caregivers’ available personal time and energy could limit effective use and integration of such services.

### Responsibilities and expectations

Family caregivers conveyed a strong sense of responsibility “to do the right thing”, though the notion of the right thing could vary. Care work had a personal symbolic and moral meaning for the caregivers, and was associated with self-worth and respect from others. There was a potential to perform care work both day and night; however, caregivers tried to balance their own responsibilities and expectations with others’ expectations, without being overwhelmed by work.

#### Family caregivers’ own expectations about care work

Family caregivers conveyed a strong sense of commitment, and viewed MND as an exceptional challenge. A bereaved caregiver described the situation as “the worst illness imaginable”, and commented:

*“And for some strange reason, I found strength during that period, I even slept well. I thought: ‘This is my life; I just have to make the best out of it … and be strong’. And I think I handled it really, really well, actually.”* (Participant 6, bereaved caregiver, trajectory lasted 4 years from first observed symptom)

While living with MND could be extremely demanding for family caregivers, the care work likewise contributed to a sense of fulfilling moral obligations, and the work thus has an intrinsic meaning. A husband caring for his wife said:

*“I was told all the time: ‘You are not supposed to be the carer for your wife. You’re her husband.’ I was told that a million times. But did I listen? It was the only chance she had to eat anything. She loves food, and so much of what we had together revolved around food, I still cook for her a lot…”* (Participant 18, family caregiver, 3 years since first observed symptom)

The immediate care work and efforts to maintain normality could represent intimacy and fulfilment of the role as spouse. Caregivers experienced that paid carers could compromise the intimacy and some of the purpose of the care work. An evening of cooking and sharing a meal with your partner would change meaning when substituting the partner with a paid carer.

#### Expectations from others

Family caregivers’ engagement was also clearly influenced by others’ expectations, such as those set by health professionals, the person with MND or other family members. Most persons with MND did not expect community health workers to take part in the lines of care work associated with family life; community health workers were mainly expected to give practical assistance and functional support. While some caregivers believed or had experienced that professionals did a good job and were reliable, others reported negative experiences with lack of skill and organisation. Unreliable community health care violated the needs of a dependent person with MND, which created strong expectations at home for family caregivers to be available for immediate care work.

In most cases, the person with MND and the family caregiver cooperated to adjust the workload for the caregiver, but there were examples of disagreements about the work. For example, family caregivers could experience that the person with MND and other family members refused nursing home care for a shorter or more permanent stay. A family caregiver told us:

*“… and it really gets down to the bone. I had to say: ‘Either you have to go to [the nursing home], or I have to leave’. You know. ‘Because at that moment, I was completely… I was completely exhausted.”* (Participant 14, family caregiver, 2 years since first observed symptom)

Disagreement along lines of work could draw disproportionally on time and energy, where immediate care work was one prominent example, as in the quote above. For example, some could view the illness trajectory as an exceptional period allowing for an exclusive focus on immediate care work and information-seeking, ignoring or actively avoiding the other lines of work.

## Discussion

The purpose of this study was to develop theory to help understand the complexity of caring for a person with MND, by separating out the lines of work in MND family caregiving and social norms accompanying this work. We found that caregivers were engaged in five lines of care work that could be parallel or closely interconnected: i) immediate care work; ii) seeking information and clarity about the disease; iii) managing competing obligations; iv) maintaining normality; and v) managing external resources and assistance. Some of the lines of work could start before a diagnosis was given, and carry on throughout the illness trajectory. Caregivers’ priorities were shaped by their interactions with the person with MND, available assistive devices, the development of the illness, and utilisation of paid care. Care work had a symbolic and moral meaning for caregivers, and was associated with self-worth and respect from others. Caregivers tried to balance their own expectations with those of others without being overwhelmed by care work.

Even though family caregivers experience the MND diagnosis as a shock [25], our study points out that caregiver work may start long before the diagnosis is definitely established. Seeking information may be a demanding effort for family caregivers, when dreading or not knowing exactly what to look for, in the absence of a formal diagnosis. Our study suggests that work prior to diagnosis is an important factor to incorporate into health professionals’ interpretation of caregiver strain, especially since eligibility for support is often based on a formal diagnosis.

We found that family caregivers seem to compromise their own needs in everyday life while caring for a family member. This “all in” approach may be due to an expectation that the illness trajectory will be short, and life may be put on hold. However, use of life-prolonging technology may lengthen the trajectory and increase the burden on family caregivers [7, 9]. In such cases, family caregivers and health professionals may need to redefine MND as a chronic rather than terminal illness, which entails an adjustment of expectations wherein health professionals may play an important role. Further, our findings underscore the necessity of discussing personal, relational and moral aspects of care delivery. We argue that the theory of lines of work provides a suitable framework for such endeavours.

Reviewing the current literature on family caregiving in MND, there is a call to move away from the burden and quality-of-life studies which predominate [10]. We contribute by expanding the body of theory around life-limiting illnesses. MND is a progressive illness characterised by uncertainty of what is to come [26], and ongoing change [6]. The specific care tasks change as the illness progresses [6, 7, 26]. We argue that lines of work may be markers in this rapidly changing landscape.

We argue that our typology of care work is more applicable to MND through expanding the original lines of work [22] from 3 to 5. Still, there are clear traces of the original theory in our typology. Immediate care work is both illness work and biographical work; actions directed at the illness as well as to impart meaning to oneself and others. Managing competing obligations and maintaining normality may resemble everyday work. The two lines of work may entail very similar activities, but it is important for health professionals to acknowledge that seemingly similar practices may encompass multiple meanings. The original theory was developed based on data from North America, a context with a radically different health policy to Norway. By presenting the management of external resources as a new line of work, we want to underscore that even though paid care is financially available, it still demands resources to integrate external support into the everyday lives of those at home.

### Emotional labour

Arlie Hochschild [27] wrote a seminal paper on emotional labour or emotion work, which is regarded as a defining part of care work [28]. Emotional labour relates to the effort to shape our emotions according to situational expectations. Heavy emotional labour on a daily basis has been underscored as a reality for family caregivers living with MND [26]. Lines of work represent new tools with which health professionals may support family caregivers’ emotional labour. A clear example is maintaining normality, and the accompanying emotional labour not to feel sad when dealing with a life-limiting and progressive illness. Immediate care work may involve expectations to display emotions of gratitude, affection and love. Managing external resources involves discussing the prognostic outlook and current care needs, even if standards or expectations are not met. Seeking information and clarity involves several emotional labour tactics, whereby family caregivers screen information and couch the information given to other family members; this is work to control emotion as well as information. When managing competing obligations, such as being together with children or performing household duties, it can be hard work to prevent emotions from becoming too overwhelming and to dictate the situation. We argue that the lines of work are a new way for health professionals to interpret caregiving situations more clearly, and to facilitate support to alleviate family caregivers’ emotional labour.

### MND caregiving and lines of work

A burden for family caregivers is the need to be available at all times, and the lack of time to oneself [10]. We found handling of immediate care work particularly challenging for family caregivers, especially when there was a lack of collaboration and support to limit this type of work as the illness unfolded. The concept illustrates challenges when utilising supportive services for family caregiving, namely the intrinsic value in the relationship, the multiple meanings involved in care work, emotional labour and a sense of being bound to certain physical spaces. Ray and Street [19] underscore the importance of negotiating and building trust for health professionals to perform intimate tasks; we add that those tasks that are not intimate can also contain symbolic meaning, constraining uptake of supportive services. This seems to be a consequence of the ambiguous or multiple meanings of immediate care work. There is evidence that family caregivers’ needs are not met effectively [29, 30], and we point out that not only constraints in primary care and among health professionals contribute to this, but also the nature of MND care itself and how this care work is handled by family caregivers.

### Implications for practice

Refusing assistance and health services may be a deliberate strategy among some family caregivers. However, it is important to acknowledge the interactional character of people – who it is performing what kind of work. Family caregivers may have difficulties voicing their own needs, especially if there is disagreement at home. Previous research suggests that an increased carer burden is closely related to the type of relationship between the person with MND and the family caregiver [11, 31–33]. Many of those with MND have symptoms of cognitive impairment [34], and the person with MND’s capacity to understand caregivers’ needs and conflicting obligations may be diminished. Some people with MND engage with services “on their own terms” [20] as a way of exerting control over the illness. However, our findings suggest that this may lead to underutilisation of services for family caregivers. We find that persons with MND and family caregivers may in some cases have conflicting needs. Health professionals need to be conscious of how they influence such conflicts.

### Methodological considerations

The participants were recruited by health professionals in MND-clinics, which may have led to a sample skewed towards family caregivers who were willing to share their experiences and who were not in conflict with health professionals. Also, next of kin who were not engaged in care are underrepresented in our sample. Although recruitment went through health professionals, the interviewer was a social scientist trained in qualitative interviewing, with a clear role as someone who could not influence (e.g. reduce) service access. Further, the variation in the sample is considerable with regard to age, gender and socioeconomic background and the sample is from three different hospital catchment areas, ranging from urban to rural parts of Eastern Norway.

We suggest that the lines of work we identify may be transferable to other diseases and palliative care settings, where there are rapidly changing care needs and advanced care work taking place at home. However, MND is expected to progress rapidly, and expectations may be different with other long-term conditions [35]. The normative landscape may be affected by whether there is a private or public health system, and how generous it is. In countries without a publicly financed health service, one may expect that expectations surrounding family carers are different.

## Conclusion

In a changing and potentially chaotic situation for family caregivers, we conceptualised their efforts along lines of work. In this perspective, care work started before a diagnosis was given, continuing throughout and potentially also beyond the illness trajectory. An array of different and changing tasks comprised the lines of work, and seemingly unimportant tasks could entail strong symbolic and moral meaning for caregivers. Caregiving was a balancing act between expectations from themselves and others and not becoming overwhelmed. Each family caregiver accommodates the lines of work into his or her local context in a unique way, and this paper has provided a framework to inform health and social workers’ discretionary judgement in MND cases.

## References

[1] Riedel M (2012). Financial support for informal care provision in Wuropean countries: a short overview. Health Ageing Newsl.

[2] Courtin E, Jemiai N, Mossialos E (2014). Mapping support policies for informal carers across the European Union. Health Policy.

[3] Fujisawa R, Colombo F. The long-term care workforce: overview and strategies to adapt supply to a growing demand. Paris: OECD Publishing; 2009. Contract No.: 44.

[4] Andersen PM, Abrahams S, Borasio GD, de Carvalho M, Chio A, Van Damme P (2012). EFNS guidelines on the Clinical Management of Amyotrophic Lateral Sclerosis (MALS) – revised report of an EFNS task force. Eur J Neurol.

[5] Kiernan MC, Vucic S, Cheah BC, Turner MR, Eisen A, Hardiman O (2011). Amyotrophic lateral sclerosis. Lancet.

[6] King SJ, Duke MM, O’Connor BA (2009). Living with Amyotrophic Lateral Sclerosis/Motor Neurone Disease (ALS/MND): decision-making about ‘ongoing change and adaptation’. J Clin Nurs.

[7] Lerum SV, Solbrække KN, Holmøy T, Frich JC (2015). Unstable terminality: negotiating the meaning of chronicity and terminality in motor neurone disease. Sociol Health Illn.

[8] Foley G (2011). The complexity of care in amyotrophic lateral sclerosis. Amyotroph Lateral Scler Frontotemporal Degener.

[9] Foley G, Timonen V, Hardiman O (2014). Acceptance and decision making in amyotrophic lateral sclerosis from a life-course perspective. Qual Health Res.

[10] Aoun SM, Bentley B, Funk L, Toye C, Grande G, Stajduhar KJ (2013). A 10-year literature review of family caregiving for motor neurone disease: Moving from caregiver burden studies to palliative care interventions. Palliat Med.

[11] Rabkin JG, Albert SM, Rowland LP, Mitsumoto H (2009). How common is depression among ALS caregivers? A longitudinal study. Amyotroph Lateral Scler Frontotemporal Degener.

[12] Bruletti G, Comini L, Scalvini S, Morini R, Luisa A, Paneroni M (2015). A two-year longitudinal study on strain and needs in caregivers of advanced ALS patients. Amyotroph Lateral Scler Frontotemporal Degener.

[13] O’Brien MR, Whitehead B, Jack BA, Mitchell JD (2012). The need for support services for family carers of people with motor neurone disease (MND): views of current and former family caregivers a qualitative study. Disabil Rehabil.

[14] Chio AM, Gauthier AP, Vignola AP, Calvo AM, Ghiglione P, Cavallo EM (2006). Caregiver time use in ALS. Neurology.

[15] Aoun SM, Connors SL, Priddis L, Breen LJ, Colyer S (2012). Motor Neurone Disease family carers’ experiences of caring, palliative care and bereavement: An exploratory qualitative study. Palliat Med.

[16] Locock L, Ziebland S, Dumelow C (2009). Biographical disruption, abruption and repair in the context of Motor Neurone Disease. Sociol Health Illn.

[17] Burke T, Elamin M, Galvin M, Hardiman O, Pender N (2015). Caregiver burden in amyotrophic lateral sclerosis: a cross-sectional investigation of predictors. J Neurol.

[18] O’Brien MR, Whitehead B, Murphy PN, Mitchell JD, Jack BA (2012). Social services homecare for people with motor neurone disease/amyotrophic lateral sclerosis: Why are such services used or refused?. Palliat Med.

[19] Ray RA, Street AF (2011). The dynamics of socio-connective trust within support networks accessed by informal caregivers. Health.

[20] Foley G, Timonen V, Hardiman O (2014). Exerting control and adapting to loss in amyotrophic lateral sclerosis. Soc Sci Med.

[21] Foley G, Timonen V, Hardiman O (2014). Understanding psycho-social processes underpinning engagement with services in motor neurone disease: a qualitative study. Palliat Med.

[22] Corbin JM, Strauss AL (1988). Unending work and care : managing chronic illness at home.

[23] Ringard Å, Sagan A, Sperre Saunes I, Lindahl AK (2013). Norway: health system review. Health Syst Transit.

[24] Kvale S, Brinkmann S (2009). Interviews: Learning the craft of qualitative research interviewing.

[25] Brown JB (2003). User, carer and professional experiences of care in motor neurone disease. Prim Health Care Res Dev.

[26] Ray RA, Street AF (2007). Non-finite loss and emotional labour: family caregivers’ experiences of living with motor neurone disease. J Clin Nurs.

[27] Hochschild AR (1979). Emotion Work, Feeling Rules, and Social Structure. Am J Sociol.

[28] James N (1992). Care = organisation + physical labour + emotional labour. Sociol Health Illn.

[29] Brown JB, Lattimer V, Tudball T (2005). An investigation of patients and providers views of services for motor neurone disease. Br J Neurosci Nurs.

[30] Van Teijlingen ER, Friend E, Kamal AD (2001). Service use and needs of people with motor neurone disease and their carers in Scotland. Health Soc Care Community.

[31] Rabkin JGP, Wagner GJP, Del Bene M (2000). Resilience and distress among amyotrophic lateral sclerosis patients and caregivers. Psychosom Med.

[32] Olsson AG, Markhede I, Strang S, Persson LI (2010). Well-being in patients with amyotrophic lateral sclerosis and their next of kin over time. Acta Neurol Scand.

[33] Olsson Ozanne AG, Strang S, Persson LI (2011). Quality of life, anxiety and depression in ALS patients and their next of kin. J Clin Nurs.

[34] Chiò A, Vignola A, Mastro E, Giudici AD, Iazzolino B, Calvo A (2010). Neurobehavioral symptoms in ALS are negatively related to caregivers’ burden and quality of life. Eur J Neurol.

[35] Røthing M, Malterud K, Frich JC (2014). Balancing needs as a family caregiver in Huntington’s disease: a qualitative interview study. Health Soc Care Community.

